# Plant-microbe interactions in the rhizosphere for smarter and more sustainable crop fertilization: the case of PGPR-based biofertilizers

**DOI:** 10.3389/fmicb.2024.1440978

**Published:** 2024-08-08

**Authors:** Monica Yorlady Alzate Zuluaga, Roberto Fattorini, Stefano Cesco, Youry Pii

**Affiliations:** Faculty of Agricultural, Environmental and Food Sciences, Free University of Bolzano, Bolzano, Italy

**Keywords:** beneficial bacteria, crop nutrition, nitrogen biofertilizer, phosphate biofertilizer, bacterial siderophores

## Abstract

Biofertilizers based on plant growth promoting rhizobacteria (PGPR) are nowadays gaining increasingly attention as a modern tool for a more sustainable agriculture due to their ability in ameliorating root nutrient acquisition. For many years, most research was focused on the screening and characterization of PGPR functioning as nitrogen (N) or phosphorus (P) biofertilizers. However, with the increasing demand for food using far fewer chemical inputs, new investigations have been carried out to explore the potential use of such bacteria also as potassium (K), sulfur (S), zinc (Zn), or iron (Fe) biofertilizers. In this review, we update the use of PGPR as biofertilizers for a smarter and more sustainable crop production and deliberate the prospects of using microbiome engineering-based methods as potential tools to shed new light on the improvement of plant mineral nutrition. The current era of omics revolution has enabled the design of synthetic microbial communities (named *SynComs*), which are emerging as a promising tool that can allow the formulation of biofertilizers based on PGPR strains displaying multifarious and synergistic traits, thus leading to an increasingly efficient root acquisition of more than a single essential nutrient at the same time. Additionally, host-mediated microbiome engineering (HMME) leverages advanced omics techniques to reintroduce alleles coding for beneficial compounds, reinforcing positive plant-microbiome interactions and creating plants capable of producing their own biofertilizers. We also discusses the current use of PGPR-based biofertilizers and point out possible avenues of research for the future development of more efficient biofertilizers for a smarter and more precise crop fertilization. Furthermore, concerns have been raised about the effectiveness of PGPR-based biofertilizers in real field conditions, as their success in controlled experiments often contrasts with inconsistent field results. This discrepancy highlights the need for standardized protocols to ensure consistent application and reliable outcomes.

## 1 Introduction

It is now a fact that the global request of agricultural food products is progressively rising because of the increase in the World population. In fact, as far as FAO projections are considered, human population inhabiting the planet is expected to reach more than 10 billion people by 2070 (Daniel et al., 2022). In this scenario, the agricultural sector is urged to increase crops’ yield to meet the standard of food security. Indeed, since the green revolution (middle of 20th century), the increased inputs of chemical fertilizer proved itself as an efficient strategy to enhance agricultural productivity (Fasusi et al., 2021). Unfortunately, due to the limited nutrient use efficiency, crops are able to exploit only a portion of the applied fertilizers, while the remaining part (generally much more than half) remains in the soil being then, very often, transformed in not available forms through different mechanisms (e.g., precipitation, adsorption, leaching, volatilization) (Zhang et al., 2018). It is evident that this phenomenon, in addition to be less sustainable, particularly in a long period vision, poses serious risks for both the environment and human beings. In this regard as well as in the framework of the 17 sustainable development goals launched by the EU,^1^ the implementation of innovative solutions for a more sustainable agriculture appears to be mandatory in the coming years. In this context, the exploitation of the potentialities lying in the so termed *rhizosphere management* seems extremely promising. The concept *rhizosphere management* refers to a series of actions aimed at increasing in this specific volume of soil the available fractions of the different nutrients for an equilibrate crop development and, then, production (Zia et al., 2021; Kumawat et al., 2022). Interestingly, beneficial soil microorganisms, also accounted as plant growth-promoting rhizobacteria (PGPR), are known to be a promising tool in this sense thanks to their capability to significantly ameliorate the edaphic conditions of the rhizosphere soil (Pii et al., 2015a). In this regard, it should be highlighted that for crops a more balanced availability of nutrients, specifically in the rhizosphere, is not only strategic to guarantee the production (food security) in a more sustainable way, but also crucial for both the quality of primary production (food safety) (Tomasi et al., 2015; Sambo et al., 2019) and the composition of its epiphytic microbial community (Valentinuzzi et al., 2021), being this latter fundamental for the post-harvest characteristics of the edible plants tissues and their shelf life.

In a previous review, we have already postulated the need of deepening our understanding about the molecular and biochemical mechanisms underpinning plant-microbe interactions (Pii et al., 2015a) as a condition for a possible wider and more effective use of PGPRs in agriculture, particularly in a context of *precision agriculture* and its greater environmental sustainability. In these last years, many PGPR strains have been isolated and characterized for their potential usefulness in the management of the rhizosphere (Fasusi et al., 2021). It is interesting to highlight that some of them are nowadays commonly used at the field scale as biofertilizers to achieve the objectives of higher food security and greater agricultural sustainability. Moreover, the results achievable with the application of these biofertilizers fit perfectly into the concept of a *smart agriculture* characterized by higher crop resilience and low external inputs that, even when essential, can be also tailored to the actual needs of the crops and to the variability of the agricultural land area (Cesco et al., 2023).

Thus, considering recent scientific advancements in microbiology and biotechnology, this review incorporates insights from 158 relevant research papers published between 1996 and 2024. Notably, the majority of these papers (84%) were published in the last decade (2014–2024), reflecting the latest developments in the field. Accordingly, the review is organized into sections that highlight the crucial roles of plant-PGPR interactions in crop fertilization. It begins with a comprehensive exploration of PGPR as biofertilizers, focusing on their ability to enhance soil fertility and crop productivity by improving nutrient availability. The review then examines how essential nutrients (e.g., N, P, K, S, Zn, Fe) are mobilized and used by plants, discussing innovative biological strategies to enhance their availability, nutrient roles in plant metabolism, their soil forms, and uptake mechanisms. Additionally, the review highlights the potential of PGPR-based biofertilizers to boost nutrient availability and plant growth. It emphasizes the successes and challenges of these biofertilizers, underscoring the need for technologies like SynComs and genetic engineering to develop high-performing biofertilizers and engineered plants for enhanced nutrient acquisition and improved soil conditions. Finally, the review addresses skepticism about PGPR-based biofertilizers due to inconsistent field results compared to controlled experiments, and it calls for standardized protocols to ensure consistent application and reliability, facilitating their transition to practical agriculture.

In a view of an ever more efficient and eco-friendly agriculture, this research is especially important for farmer communities committed to sustainable agriculture systems. Adopting PGPR-based biofertilizers can enhance nutrient use efficiency, minimize environmental impacts, and boost both crop yield and quality (Kumawat et al., 2023). This research supports the development of innovative and sustainable agricultural methods that address increasing food demand while protecting the environment and ensuring the long-term viability of farming operations.

## 2 Plant growth-promoting rhizobacteria as biofertilizers

About 20 years ago, the term biofertilizer was meant to define “a substance containing living microorganisms which, when applied to seeds, plant surfaces, or soil, colonizes the rhizosphere or the interior of the plant and promotes growth by increasing the supply or availability of primary nutrients to the host plant” (Vessey, 2003). Through the years, several pieces of research have been carried out and, thanks to the knowledge acquired, one of the most recent definition, that is more in line with the content of this review, describes biofertilizer as “a product containing beneficial microorganisms with the potential to improve soil fertility and crop productivity by enhancing nutrient availability” (Atieno et al., 2020). Since PGPR have been broadly reported as beneficial bacteria, they have been used worldwide as biofertilizers and characterized for their beneficial properties. The most reported effects are mainly ascribed to the involvement of members of the genera *Azospirillum*, *Rhizobium*, *Bacillus*, *Pseudomonas*, *Burkholderia*, *Enterobacter*, *Herbaspirillum*, *Bradyrhizobium*, *Pantoea*, *Paenibacillus*, *Azotobacter*, and *Serratia* (Tabassum et al., 2017; Bhadrecha et al., 2023; Kumawat et al., 2024). However, to be considered an ideal biofertilizer with the potential to enhance plant nutrition and development upon inoculation, a PGPR strain should present the following characteristics:

1. It should possess a high rhizosphere colonization rate upon inoculation (Backer et al., 2018).
2. It should be able to establish a stable and long-term colonization with the host plant (Liu et al., 2019).
3. It should show a maximal, consistent and reproducible efficacy under a range of field conditions (Herrmann and Lesueur, 2013).
4. It should be able to face intense competition with indigenous soil microbes (Backer et al., 2018).
5. It should promote plant growth by improving nutrient availability at rhizosphere level (Aloo et al., 2022), similarly to a localized fertilization.
6. It should present broad-spectrum versatility that encompasses a wide range of environmental adaptations (Mitter et al., 2021).
7. It must be environmentally friendly and consistent with the requirements of sustainable practices (Keswani et al., 2019).
8. It should be classified as safe, posing low threat to human health, soil and live communities (Keswani et al., 2019) as desired in a context of One Health.

It is interesting to note that PGPR can also directly promote plant growth by providing phytohormones or signaling molecules, or they can indirectly influence plant health by synthesizing bioactive compounds with potential as antimicrobials or stress tolerance, hence earning the designation of *biostimulants* (Kaushal et al., 2023). These compounds can modulate the root system architecture development, improve the photosynthetic capacity of a plant, or activate the antioxidant defense system (Rosier et al., 2018; Grover et al., 2021). However, since the focus of this review is primarily on PGPR that can turn sparingly available fractions of essential nutrients into more accessible forms for plants, PGPR with *biostimulating* actions will not be considered and discussed.

For this reason, PGPR-based biofertilizers can be classified based on the specific mineral nutrient they contribute to promote the availability for plants (Table 1). For instance, some PGPR strains are known to facilitate different processes including nitrogen (N) fixation (i.e., transforming atmospheric N_2_ into NH_3_ or NH_4_^+^), solubilization of nutrients (i.e., turning insoluble forms of P into H_2_PO_4_^−^, insoluble K pools into the free ionic form [K^+^], sparingly available Zn sources into the free ionic species [Zn^2+^]), oxidation of substrates (i.e., producing SO_4_^2−^ via oxidation of S^0^-containing sources), and/or metal chelation/complexation (e.g., exuding siderophores, phenolic compounds and/or organic acids to scavenge Fe^3+^ and other nutrients) (Colombo et al., 2014; Malusá and Vassilev, 2014; Mitter et al., 2021; Aloo et al., 2022). Moreover, it is important to mention that some strains of PGPR acting as biofertilizers, may have the ability to increase at the same time the supply to plants of more than one essential nutrient. Therefore, it is interesting to highlight that the action of these bacteria, whether single- or multi-element, is specifically localized in the rhizosphere, making them an extremely valuable tool for a precision agriculture context. In fact, the increasingly chance to use the endogenous nutritional resources of soil locally significantly reduces the need to broadcast synthetic fertilizers (Vacheron et al., 2013; Pii et al., 2015a, 2016; Rosier et al., 2018). Furthermore, neglecting or not giving appropriate attention to the PGPR strains that exert direct effects on crop growth and health, particularly in the context of more efficient and more sustainable agriculture, would certainly represent a missed opportunity.

**Table 1:** Classification of biofertilizers based on the mineral nutrient supplied.

Type	Mechanism
N-fixing PGPR	Symbiotic bacteria (e.g., *Rhizobium*), associative bacteria (e.g., *Azospirillum*) or free-living bacteria (e.g., *Azotobacter*) fixing atmospheric nitrogen (N_2_) by means of the nitrogenase enzyme and producing NH_3_/NH_4_^+^ (Bhadrecha et al., 2023)
P-solubilizing PGPR	Bacteria converting, through a ligand-exchange mechanism based on the organic acids, hydroxyl ions and CO_2_ released, barely available soil sources of P into plant available forms (e.g., H_2_PO_4_^−^) (Bhadrecha et al., 2023)
K-solubilizing PGPR	Bacteria solubilizing K from K-bearing minerals and enhancing its free ionic form in soil solution via the excretion of organic acids (Mitter et al., 2021)
S-oxidizing PGPR	Bacteria releasing in soil SO_4_^2−^ after the oxidation of elemental S (S^0^)-containing substrates as a source of energy (Mitter et al., 2021)
Zn-solubilizing PGPR	Bacteria releasing into the rhizosphere organic ligands with enhanced ionic Zn^2+^ solubility and metal complexation (Kamran et al., 2017)
Siderophore-producing PGPR	Bacteria releasing chelating compounds with scavenging properties of metal (like Fe, Cu, Mo, Mn, Co, and Ni and other essential elements) from barely available sources (Ahmed and Holmström, 2014)

## 3 Plant nutrient acquisition in a context of plant-biofertilizer interaction

### 3.1 The case study of nitrogen

**Figure 1:**
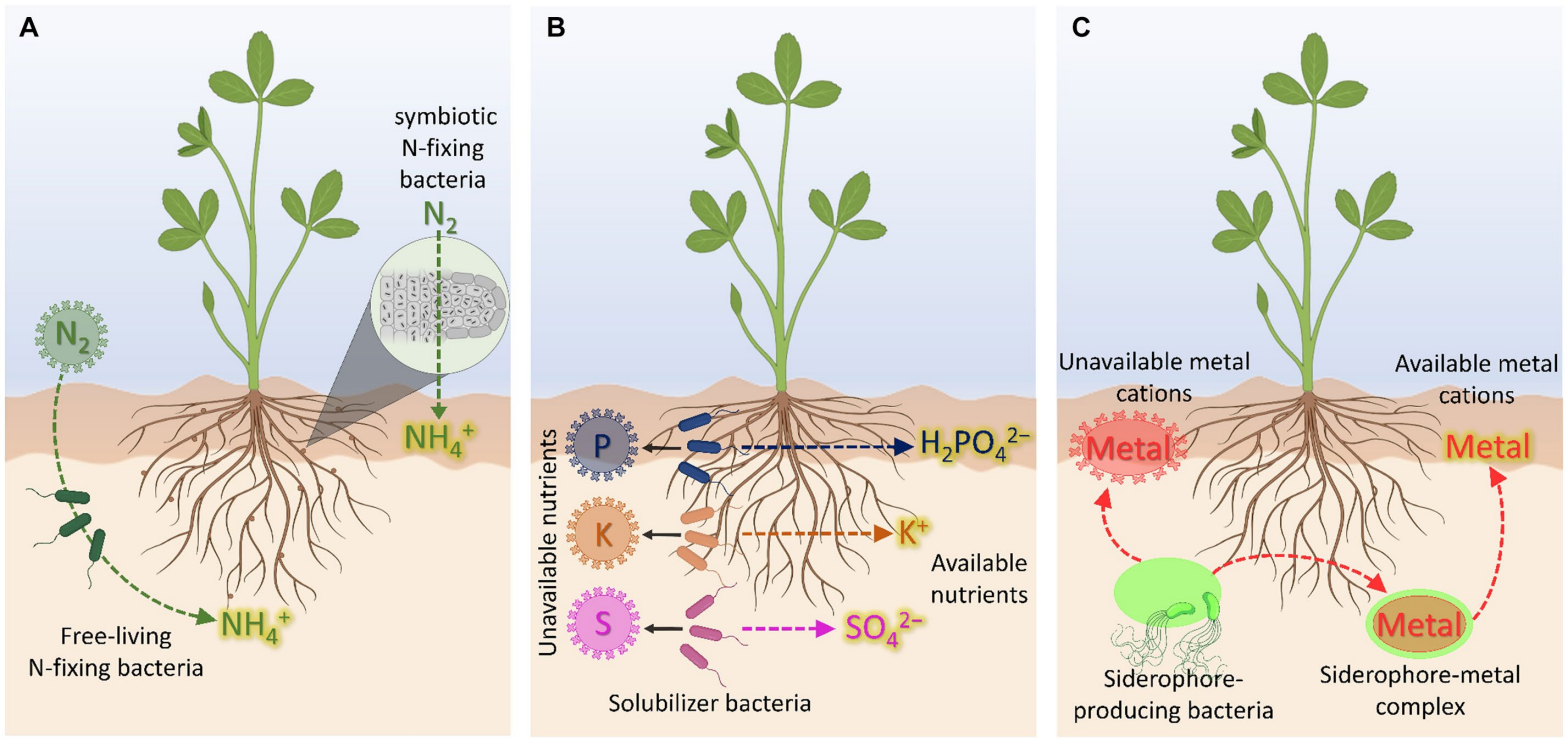
Biofertilizer-mediated nutrient availability. **(A)** N-biofertilizers. **(B)** P-, K-, and S-biofertilizers. **(C)** Cation-chelating biofertilizers based on siderophore-producing bacteria. Created with BioRender (https://biorender.com/).

Nitrogen, accounting for 1–5% of the whole plant dry biomass, is a key component of amino acids, proteins, and chlorophyll being, therefore, essential for plant growth and development (Jiang et al., 2020). The largest reservoir of N in the world is found in the atmosphere, mostly as nitrogen gas (N_2_), which can only be used by plants after transformation through chemical [via lightning action, CNF, (Barth et al., 2023)] or biological N_2_ fixation (BNF). This latter process can be performed by diazotrophs prokaryotes (N_2_ fixers), including symbiotic bacteria, as well as free-living or associative bacteria (Figure 1A). These bacteria encompass *nif* genes encoding the nitrogenase enzyme, which catalyzes the reduction of N_2_ to NH_4_^+^ (Soumare et al., 2020). Indeed, the bacteria-derived NH_4_^+^, if available in the rhizosphere, can then be also taken up by roots through plasma membrane transporters belonging to the AMT1 subfamily of the ammonium transporter/methylammonium permease family by exploiting either NH_4_^+^-uniport or NH_3_/H^+^ co-transport (Loque and Wirén, 2004; Neuhauser and Ludewig, 2014). In this regard, it should be mentioned that pieces of evidence showing alternative routes for NH_4_^+^ movement across the plasma membrane have been gathered through the years. In particular, it was observed that NH_4_^+^ can be also taken up by plants through non-selective cation channels (NSCC) and K^+^ specific channels, as for instance AKT1 (Coskun et al., 2013; Esteban et al., 2016). Nevertheless, it is worth mentioning that these not specific mechanisms have been suggested to play a predominant role in NH_4_^+^ acquisition only when the external concentration of the nutrient is higher than 1–2 mM (Muratore et al., 2021), being thus responsible for the onset of NH_4_^+^ toxicity in plants.

The most studied symbiotic N_2_-fixing bacteria are rhizobia, which can establish symbiotic relationships with leguminous plants by forming specialized root nodule organs. Rhizobia, once enclosed in these nodules, experience a microaerobic environment, where nanomolar levels of oxygen (20–50 nM) are necessary for the *nif* gene expression and consequently, ensure high amounts of fixed N_2_ (Ledermann et al., 2021). It is estimated that the overall contribution of BNF in rhizobia-legumes systems is between 50 and 80% accounting for at least 70 million metric tons of N fixed per year (Sindhu et al., 2019). For this reason, highly effective strains of rhizobia such as *Rhizobium*, *Bradyrhizobium*, and *Sinorhizobium* are often used as biofertilizers in most major legume crops thanks to their ability to fix N_2_ in amounts as high as the amounts provided by synthetic N-fertilizers. In 2022, the global biofertilizer market was valued at USD 2.67 billion, whereby USD 1.92 billion was represented rhizobia-based biofertilizers, while the remaining USD 750 million constituted biofertilizers based on free-living N-fixing bacteria, P-solubilizing bacteria, K-solubilizing bacteria, and other PGPR. Moreover, their demand is estimated to grow by a further 12.3% compounding annual growth rate (CAGR) to reach about USD 6.97 billion by 2030.^2^

Differently of symbiotic rhizobia, free-living associative bacteria inhabit the surfaces or the interstitial spaces of the host plant and use the root exudates as carbon sources to fix N_2_ for their own use, providing the excess to the host (Guo et al., 2023). Several free-living N-fixing bacteria, including members from the genera *Azospirillum*, *Azotobacter*, *Herbaspirillum*, *Bacillus*, *Clostridium*, *Burkholderia*, and *Gluconobacter,* have been reported to fix N_2_ in the rhizosphere and bulk soil. These bacteria are particularly important for N nutrition in non-leguminous plants. Nonetheless, their contribution on BNF is more limited, providing up to 25% (13–22 kg N ha^−1^ yr.^−1^) of the total N in crops like rice, wheat and maize (Ladha et al., 2016). In this context, an attractive avenue to improve BNF in non-leguminous crops has been the use of genetic approaches to engineer root nodulation with symbiotic bacteria also in cereals. However, none of the attempts performed to date has succeeded (Pankievicz et al., 2019). On the other hand, notably progress has been made in genetic engineering strategies toward better N-fixing bacteria. Thus, by understanding the heterologous expression of the *nif* gene cluster in *Klebsiella oxytoca*, *Azotobacter vinelandii*, and *Paenibacillus polymyxa*, many scientists have been able to transfer their N-fixation trait to non-diazotrophic hosts (Li and Chen, 2020). For instance, the metabolic engineering of *A. vinelandii* resulted in a constitutive expression of *nif* genes with an increased production of NH_4_^+^. Thanks to this improvement, an enhanced growth of not N-fertilized cucumber plants has been recorded in plants when root colonized by the engineered *A. vinelandii* strain (Ambrosio et al., 2017). It is interesting to highlight that increased concentrations of NH_4_^+^ were also reported by (Pii et al., 2019) in the association between *Azospirillum brasilense* and maize plants. The authors also described that *Azospirillum* counteracted the plant response to NO_3_^−^ uptake as a result of the activation of processes linked with NH_4_^+^ fluxes and assimilation.

Recently, Rago et al. (2019) proposed to use N-fixing bacteria in association with electrochemical systems for the reduction of N_2_ to NH_4_^+^ at low energy cost, in a process called bioelectrochemical N fixation (e-BNF). This process aims at the production of microbial biomass through simultaneous N_2_ and CO_2_ fixation using electricity as only energy input, thus opening a new path toward local on-demand N-biofertilizer production, based on renewable power. In this regard, it is worth to mention that by using genetic manipulation in combination with e-BNF, (Dong et al., 2021) integrated a modified *nif* gene cluster into the genome of the non-diazotrophic cyanobacterium *Synechococcus elongatus* and, thereafter an e-BNF system was used for NH_4_^+^ production. The authors reported that the e-BNF led to an increased production of NH_4_^+^ which was 21 times higher than that generated from solely photosynthesis-driven N fixation. Accordingly, these strategies provide novel technological feasibility to raise BNF inputs while alleviating the negative impacts of synthetic N-fertilizers (e.g., low crop N-efficiency, leaching, volatilization, use of not renewable energy for the synthesis).

### 3.2 The case study of phosphorus

Phosphorus is an essential nutrient representing the 0.1–0.8% of the dry matter and playing crucial roles in photosynthesis, energy transfer, respiration, and biosynthesis of molecules (Vance et al., 2003). In soil, P is present in both inorganic (i.e., free ionic species and minerals) and organic forms (i.e., phosphates monoesters and diesters, polyphosphates, and phosphonates), being the mineral and organic fractions not usable by plants as they are (Ducousso-Détrez et al., 2022). Moreover, it should be noted that the free ionic species have a strong affinity for soil particles being, therefore, readily retained in soil. As a consequence, only a minimal part of P in soil (about the 0.1%) is available for plant acquisition (Lambers, 2022). At root level, P is taken up as inorganic phosphate (H_2_PO_4_^−^/HPO_4_^2−^, Pi) against the electrochemical gradient established at the plasma membrane (Hinsinger, 2001). The acquisition of Pi from the soil solution is mediated by high affinity transporters, encoded by the gene family *PHT1* (Naureen et al., 2018), whose energy demand is sustained by the activity of plasma membrane H^+^-ATPase (Młodzińska and Zboińska, 2016). On the other hand, it is well known that, with respect to the amount of P commonly supplied to a crop with chemical fertilizers, the majority undergoes precipitation and leaching processes, limiting then the fraction actually used by the crop to only 10–20% (Ibrahim et al., 2022). In this context, the use of P-solubilizing bacteria (PSB) can nowadays represent a valid opportunity not only for an efficient mining and solubilization of P sources endogenously present in soil but also to limit the application of fertilizers characterized by such low use efficiency (Figure 1B). Moreover, if the localized effect of the strain (depending essentially on the root colonization by the bacteria) is considered, the sustainability of the entire process appears even more evident.

From the point of view of the mechanisms, the P solubilization mediated by PSB include the production and release of organic acids, phenolic compounds, siderophores, hydroxyl ions, protons, and CO_2_ leading to soil acidification and/or ion exchange of P by carboxylates (Alori et al., 2017). In fact, significant advancements including the isolation and characterization of bacterial genes have demonstrated that the glucose dehydrogenase (GDH, encoded by the *gcd* gene) and its cofactor pyrroloquinoline quinone (PQQ, encoded by the *pqq* operon) mediate the secretion of gluconic acid associated with P solubilization in Gram-negative bacteria (Rasul et al., 2019). Other mechanisms governing the inorganic P-solubilization by PSB include the production of hydrolytic enzymes encoded by the pyrophosphatase (*ppa*) and exopolyphosphatase (*ppx*) genes (Wu et al., 2022). However, with respect to the component of the mechanism connected with the organic acids exudation, it has been recently demonstrated, by using Leonard’s jars and *Enterobacter* sp., that the organic acids mainly involved in a soil–plant system (i.e., oxalic and citric acids) did not correspond to those released by the same bacterium in the highest concentrations under *in vitro* conditions (i.e., ketoglutaric and malic acids) (Zuluaga et al., 2023b). This experience clearly shows that the combination of the information acquired in *in vitro* experiments with that in conditions closer to the cultivated soil (i.e., *in vivo*) is crucial for the identification of the bacteria with the best P-solubilizing attributes for the field level application (Elhaissoufi et al., 2023).

Once solubilized, the crops benefit from the increased P availability enhancing their total biomass accumulation and productivity (Zuluaga et al., 2021; Wang and Song, 2022; Elhaissoufi et al., 2023). A large number of PSB has been described in this sense, including species of *Burkholderia*, *Enterobacter*, *Bacillus*, *Pseudomonas*, *Rhizobium*, *Ralstonia*, *Serratia*, *Azotobacter, Paenibacillu*s, and *Erwinia* (Zuluaga et al., 2020; Timofeeva et al., 2022). However, to better exploit the abilities of these bacteria, it becomes necessary a deeper understanding of their modes of action (including the regulation and elicitation of the interesting traits) and the development of strategies capable to boost their biofertilization properties. In this scenario, considering that the repeated (year after year) application of P fertilizers has led in the long period to a P accumulation in many agricultural soils (also known as legacy P, Doydora et al., 2020), the current strategies are focusing on the ability of PSB to mobilize legacy P supplying the nutrient to plants, either alone or in combination with external P inputs. The results have shown that PSB can enhance P uptake of plant by increasing the immediately plant-available P pool in rhizosphere soil (Long and Wasaki, 2023). However, the supply of limited doses of exogenous P can potentiate the effect of PSB by solubilizing higher amounts of endogenous native Ca-, Fe- and Al-bound P (Alam et al., 2023). The need for supplemental P with PSB is expected, since these bacteria also require P and other essential elements for their own metabolic processes (Doydora et al., 2020).

Finally, it is important to mention what highlighted in a meta-analysis conducted by (Zutter et al., 2022) considering 104 articles published between April 1976 and May 2021 related to “phosphate solubilizing bacteria” and “plant growth.” The authors raised the following issues:

- biomass is generally chosen as the best indicative parameter to evaluate a plant P-status. However, the meta-analysis showed that plant growth promotion is not always related to improved P-uptake. Therefore, the use of multiple imaging techniques could also be implemented as a useful tool to assess P supply by PSB;
- the use of PSB consortia does not significantly provide an added benefit to plant growth and P-uptake compared to single strains. Thus, the combination of several PSB should be designed with care;
- strains of *Burkholderia* sp. and *Enterobacter* sp. have shown better effects on plant P-uptake and biomass than *Bacillus* sp. and *Pseudomonas* sp., but the use of these two strains is restricted due to their danger to human health. However, it has been assured that some species among these genera are unlikely to harm humans and, therefore, they should be used as safe P-biofertilizers in agriculture (Sawana et al., 2014).

With respect to biotechnology application, few experiences of bioengineering PSB are described in literature. For instance, the incorporation of a *pqq* gene cluster in *Herbaspirillum seropedicae* resulted in the activation of gluconic acid secretion, which conferred the ability of P solubilization and the improvement of N and P status in inoculated rice plants (Wagh et al., 2014, 2016). In addition to its intrinsic ability to solubilize inorganic P, the phosphobacterium 9320-SD was transformed with a bacterial gene encoding phytase and this allowed the mineralization of organic phosphate into plant-available P (Liu et al., 2015). In this regard, the manipulation of key genes through genome editing approaches could be a good strategy for enhancing the potential of PGPB as biofertilizers. Nonetheless, oncoming investigations should also include field trials under variable conditions to assess the real potential of genetically modified bacterial strains.

### 3.3 The case study of potassium

Potassium, ranging from 0.5 to 6% of the plant dry matter, is the third more represented essential nutrient in plants and it is implicated in crucial metabolic functions including photosynthesis, transport of nutrients and photosynthates, ATP production, and protein synthesis (Szczerba et al., 2009; Hasanuzzaman et al., 2018). In soil, despite being one of the most abundant elements, only 2–3% is directly available for plant uptake in the free ionic form K^+^ (Olaniyan et al., 2022). At root level, K^+^ uptake is achieved by the activity of High Affinity Transport Systems (HATS) and Low Affinity Transport Systems (LATS) (Britto and Kronzucker, 2008). The high-affinity K^+^ uptake is achieved through the activity of either the KT/HAK/KUP transporter family or the HKT transporter family when the external concentration of K^+^ is in the μM range (Santa-Maria et al., 2000). In this case, K^+^ is taken up exploiting a symport mechanism along with H^+^, in an energy-dependent process (Maathuis et al., 1997). As in the case of other mineral nutrients, the proton motive force across the plasma membrane is generated by the activity of ATPase enzymes, which hydrolyze ATP to extrude H^+^ toward the rhizosphere (Palmgren, 2001; Pardo et al., 2006). Conversely, the LATS is active when the external K^+^ concentration is higher than 0.3–0.5 mM (Wang and Wu, 2013), whereby it is taken up by non-selective and selective ion channels, as in the case of AKT1 transporter (Hirsch et al., 1998). With respect to the soil-microorganism-plant interface, it is well known that one of the very effective strategies to increase the K available fraction is that relying on the use of K-solubilizing bacteria (KSB, Etesami et al., 2017, Figure 1B). The action of these bacteria is rather complex and involves the release of organic acids (OAs), the secretion of exopolysaccharides (EPS), and the formation of biofilms on mineral surfaces (Sattar et al., 2019). The release of organic acids at the mineral surfaces, as well as the acidolysis and the carbonic acid production microbial respiration-dependent (Basak et al., 2017), can favor the chemical weathering of K-bearing minerals, like mica, biotite, muscovite, feldspar, illite, and orthoclase (Sattar et al., 2019). Differently, the secreted EPS can strongly adsorb organic acids, and the adhesion on the surface of K-bearing minerals will result in a locally higher concentration of OAs, with clear impact on weathering effects (Liu et al., 2012). Similarly, when biofilm formation is considered, microbial cells are concentrated within a self-produced extracellular polymers matrix (composed by proteins, DNA and polysaccharides). These conditions offer the chance to boost the biochemical reactions underpinning K mobilization (Nagaraju et al., 2017).

In this context there are several PGPR strains including *Arthrobacter*, *Bacillus*, *Burkholderia*, *Enterobacter*, *Pseudomonas*, *Acidothiobacillus*, *Flavobacterium*, and *Thiobacillus* that, exhibiting one or more of these actions at a time, can be defined as KSB bacteria (Olaniyan et al., 2022). However, although the contribution of these bacteria in improving K acquisition in crops is out of any doubt, particularly in soils with impaired fertility, it should be highlighted that in general the extent of this contribution seems to not cover the entire plant requirement for an equilibrate development (Ali et al., 2021; Aloo et al., 2022). Moreover, since the bacteria-based nutrient mobilization is generally relatively slow, the single inoculation of KSB may not be able to fulfill the nutrient plant needs. Accordingly, recent studies have shown that the use of biofertilizer bacterial consortia, composed of KSB in combination with other types of PGPR-based biofertilizers (i.e., integrating bacterial actions that are multiple, concurrent, and involving more than a single nutrient at a time), constitutes an innovative eco-friendly strategy to improve in a wider way the plant nutrient availability and agricultural production (Jain and Hari, 2021; Sherpa et al., 2021).

Although any bioengineering applications to the KSB traits has been described in the literature yet, the whole genome sequencing of potential KSB strains [e.g., *Bacillus aryabhattai* (Chen et al., 2022) and *Priestia megaterium* (Wu et al., 2023)] is increasingly being undertaken to possibly locate K solubilization-related genes that might be helpful in the development of biotechnological tools.

### 3.4 The case study of sulfur

Sulfur, representing up to 0.5% of the total plant dry biomass, is an essential macronutrient and crucial for the biosynthesis of the two S-containing amino acids (i.e., methionine and cysteine). These amino acids are then precursors for the synthesis of several other secondary metabolites (e.g., glutathione, phytochelatins, phytosiderphores) that are relevant for a balanced and healthy plant development (Astolfi et al., 2021). Even though the total amount of S in soils ranges from 19 to 4,000 mg kg^−1^, only a limited fraction represented by the inorganic sulfate form (SO_4_^2−^) is readily available for plant uptake (Ranadev et al., 2023). In fact, the 90–95% of the total S pool in soil is generally present in the organic and/or mineral forms, not directly acquirable by roots unless after mobilization/oxidation of S^0^ to SO_4_^2−^ (Mitter et al., 2021). The remaining (5–10%) is mainly represented by the relatively mobile SO_4_^2−^ fraction, whose 95% is adsorbed to the mineral soil fraction. Consequently, generally not more than 5% of the total SO_4_^2−^ is present in the soil solution ranging from 0.15 to 1.2 g kg soil^−1^ (Scherer, 2009).

At the root cells level, the SO_4_^2−^ uptake is mediated by sulfate transporters (SULTR) family, which, depending on the plant species, can be formed by 12 to 16 members, subdivided in four distinct functional groups (Takahashi et al., 2011). Functional studies in *Arabidopsis thaliana* have shown that SULTR1;1 and SULTR1;2 mediate the high affinity transport under SO_4_^2−^-limiting growth conditions (Takahashi, 2019). The uptake of SO_4_^2−^ is achieved through an active transport, whose motive force is suggested to be constituted by the proton gradient formed across the plasma membrane (Buchner et al., 2004). Based on this background it appears clear that the majority of S in soil is present in forms and/or redox states that cannot be directly used by the plant. If on the one hand these aspects reveal how strategic for successful crop production is the practice of S fertilization, on the other they highlight how much the biological S-oxidation by sulfur-oxidizing bacteria (SOB) represent an interesting opportunity for developing new biofertilizers able to exploit more efficiently the endogenous soil S-sources (Figure 1B). In this context, it should be noted that, typically, the chemolithotrophic bacteria are the most dominant and efficient S-oxidizers in soils, known for converting the reduced forms of S (i.e., S^2−^, S^0^, SO_3_^2−^, and S_2_O_3_^2−^) into SO_4_^2−^ (Tourna et al., 2014). This group comprises several species of *Thiobacillus*, *Acidithiobacillus*, *Sulfolobus*, *Thermothrix*, *Paracoccus*, *Thiomicrospira*, *Thiosphaera*, and *Acidianus* (Ranadev et al., 2023). However, the process of S oxidation can be also performed by heterotrophic bacteria belonging to the genera *Burkholderia*, *Enterobacter*, *Klebsiella*, *Pseudomonas*, and *Xanthobacter*, as described by (Chaudhary et al., 2022). Regarding the mechanisms of S oxidation by the SOB, three oxidation processes have been proposed: (1) the Sox pathway, in which photo- and chemolithotrophic alpha-proteobacteria can directly oxidize all reduced forms of S into SO_4_^2−^ (encoded by the *sox* operon); (2) the S4I pathway, where obligate chemolithotrophic beta- and gamma-proteobacteria produce S_4_O_6_^2−^ as an intermediate; and (3) the branched thiosulfate oxidation pathway, that involves intracellular sulfur depositing under anaerobic conditions (Ranadev et al., 2023). With reference to the field experiences, it has been well demonstrated with several agricultural crops that the inoculation of SOB in combination with elemental S sources promotes plant growth improving the yield (Pourbabaee et al., 2020; Ranadev et al., 2023). Furthermore, the application of elemental S enriched with SOB seems not only to speed up the elemental S conversion into SO_4_^2−^, but to induce a rhizosphere soil acidification with the consequent effects on the availability of various other macro- and micro-nutrients (Chaudhary et al., 2022; Nadeem et al., 2022).

With respect to the bioengineering applications to the interesting traits of these SOB, up to now no experiences are reported in literature. Nonetheless, several authors are carrying out efforts aimed at investigating the whole genome of SOB strains (Koch and Dahl, 2018; Watanabe et al., 2019) in order to identify key genes and functions that might be used as valuable traits for genetically engineered strains as S biofertilizers in agriculture.

### 3.5 The case study of zinc

Zinc is an essential micronutrient whose concentration levels in plant tissues varies from 20 to 100 mg kg^−1^ (shoot dry matter). It is required for plant growth (e.g., integral component of lipids, proteins and carbohydrate synthesis via carbonic anhydrase), plant development (e.g., DNA replication, RNA biosynthesis, involvement in IAA synthesis), and plant resistance against biotic and abiotic stresses (e.g., detoxification of superoxide radicals) (Hassan et al., 2020). In soil, depending on the geochemical composition and weathering of parent rocks, it is naturally present at concentrations ranging from 10 to 300 mg kg^−1^ (Kabata-Pendias and Mukherjee, 2007). Sorption, complexation and, to a lesser extent, dissolution/precipitation mechanisms determine the solid–liquid partitioning of Zn in soil. When the soluble fraction is considered, Zn can occur in different forms, either as free divalent cation (Zn^2+^) or complexed with organic ligands (Cesco et al., 2022). Although Zn^2+^ is the form mainly taken up at root level, the uptake of chelated forms (e.g., Zn-phytosiderophore or zinc-organic acids) across the plasma membrane is also reported in some plants (von Wirén et al., 1996). The Zn^2+^ transport across the root plasma membrane is predominantly mediated by members of the ZIP (Zn-regulated transporter proteins) family (Greco et al., 2012). Nevertheless, several pieces of research have also highlighted that ZIP family protein iron-regulated transporters 1 and 3 (IRT1 and IRT3) can significantly contribute to Zn influx in root cells (Dubeaux et al., 2018; Lee et al., 2021). Furthermore, members of the Yellow-Stripe1–like (YSL) family, the heavy metal ATPases (HMAs), and the cation diffusion facilitator (CDF) have also been shown to play a role in Zn homeostasis in plants (Sinclair and Krämer, 2012; Cardini et al., 2021).

The highest values of Zn are generally found in soils rich in organic matter, clay minerals, and CaCO_3_. On the contrary, soils characterized by high pH values and low organic matter contents can often induce Zn deficiency phenomena, particularly in wet and cool conditions. To prevent in crops the consequences of this shortage (which include smaller leaves, chlorosis and stunted development), the exogenous application of ZnSO_4_ as chemical fertilizer has been extensively used due to its high solubility and low cost. However, ZnSO_4_ is rapidly converted into insoluble forms in soils, and only 2–5% of the total Zn applied can be actually used by plants (Beig et al., 2023). In the frame of PGPR, Zn-solubilizing bacteria (ZnSB) are nowadays emerging as a potential eco-friendly alternative to enhance Zn availability in rhizosphere soil. Similarly to what described for PSB and KSB, the proposed mechanism underlying the ZnSB’s contribution to the Zn acquisition in crops is essentially based on a Zn-solubilizing action through a series of processes including acidification of the nearby soil (e.g., thanks to the exudation of organic acids and protons), the metal–ligand exchange (e.g., via the release of carboxylates, siderophores and other organic ligands) and/or the activity of redox systems on cell membranes (Kamran et al., 2017). In this context, several pieces of literature have described that PGPR belonging to the genera *Pseudomonas*, *Bacillus*, *Rhizobium*, *Enterobacter*, *Priestia* and *Pantoea*, when applied to different crops (cereals and legumes), have demonstrated effectiveness in improving the Zn acquisition with beneficial effects both on crop development and its productivity (Kamran et al., 2017; Yadav et al., 2022; Srithaworn et al., 2023). Moreover, there is recent evidence supporting the idea of an effectively use of ZnSB also to pursue the objective of biofortification of agricultural production, such as the case of wheat grain. In this specific case, a significant seed biofortification was obtained by using ZnSB in combination with Zn chemical fertilizers (Yadav et al., 2022; Saleem et al., 2023).

With respect to innovations through genetic modifications, there is no evidence in literature describing programs to improve the Zn-solubilizing traits of these bacteria through biotechnological approaches. Furthermore, deeper insights into their Zn-solubilizing mechanisms are still necessary to maximize Zn accessibility in the rhizosphere environment.

### 3.6 The case study of iron and other micronutrients: bacterial siderophores

Iron (Fe) belongs to the group of essential micronutrients, being, among these, the one required in highest amounts. Due to its ability to easily switch between to oxidation states (Fe^2+^ and Fe^3+^), it is a cofactor of proteins involved in electron transfer and of many enzymes catalyzing redox reactions. For these reasons, Fe is involved in important metabolic pathways like photosynthesis, respiration, chlorophyll biosynthesis, and antioxidant defense system (Murgia et al., 2022). In soil, although present at relatively high concentrations (20–40 mg kg^−1^) mainly as ferric (hydro)-oxides, its availability for plants is limited due to the poor solubility of the metal in the soil solution (Zuluaga et al., 2023a). Crops display at their root level the functionality of two different strategies of Fe acquisition: in dicots the Fe^III^-chelate reduction-based one while in monocots a strategy dependent on chelation by phytosiderophores with high affinity for binding Fe^3+^ (for more details see also Morrisey and Guerinot, 2009).

With respect to the PGPR and plant Fe nutrition, several pieces of literature have demonstrated that these bacteria can significantly enhance in plants the Fe acquisition process in scenarios of restricted Fe availability. This effect is ascribed both to the synthesis and the release in the rhizosphere of huge amounts of organic acids, phenolic compounds, siderophores (favoring essentially the component of Fe solubilization), and to an enhanced activity of the plasma membrane ferric chelate reductase (FCR) of roots (enhancing the nutrient uptake step). In this regard, it is interesting to mention the experiences gained with the root inoculation with *Azospirillum brasilense* in cucumber plants. In these specific conditions, the PGPR’s effect appears to be ascribable to a rather complex of actions including not only an enhanced exudation of chelating compounds and an induced FCR activity as previously described, but also the modulation of the expression levels of key genes related to Fe acquisition and allocation (Pii et al., 2015b, 2016; Marastoni et al., 2019). Moreover, it is worth to highlight that this beneficial effect was also evident in cucumber plants fed adequately with Fe resulting in contents of the nutrient in the tissues much higher than those observed in plants not inoculated with the PGPRs (Pii et al., 2016).

In recent years, siderophore-producing bacteria (SPB) have been proposed as a sustainable alternative to synthetic fertilizers. Bacterial siderophores are low molecular weight organic compounds exhibiting cation-chelating properties and synthesized either under nutrient shortage in order to cope with the nutritional disorder or to alleviate the toxicity level of heavy metal (Vijay et al., 2023). Although the term siderophore is more often used for the context of Fe nutrition and to indicate specifically a ligand able to form chelates with Fe, these compounds display affinity also for other elements like Cu, Mn, Mo, and Zn, all relevant for the plant and its metabolism (Figure 1C). Therefore, the root inoculation with SPB could be also envisaged as a strategy to improve the bioavailability not only of Fe but also of these other micronutrients. In this context, regarding specifically to the SPB contribution to Fe nutrition, Ferreira et al. (2019) demonstrated that a siderophore-based product prepared from *Azotobacter vinelandii* culture ameliorated Fe deficiency of soybean plants, thus representing a friendly Fe-fertilizer alternative for application in calcareous soils. Furthermore, (McRose and Baars, 2017) provided evidence that, under Fe starvation, *A. vinelandii* produced higher amounts of vibrioferrin, an α-hydroxycarboxylate siderophore. However, under Mo limitation, this bacterial strain completely repressed the production of vibrioferrin, producing instead higher concentrations of protochelin, a siderophore belonging to the catechol class. These results suggest that the SPB *A. vinelandii* can selectively direct the production of siderophores depending on the element for which the limitation is sensed. However, it is important to highlight that the stability of the complex metal-siderophore (Me-S) can play a strategic role, both in a negative and positive sense. In fact, if on the one hand, when sufficiently strong, the Me-S complex stability ensures its persistence in the soil even in particularly disadvantaged conditions, on the other, when excessively strong, could represent a limit to its use by the roots, in particular for the strategy based on the ligand exchange like in monocots. In this regard it should be highlighted that the scientific debate is still wide (Colombo et al., 2014). Therefore, for a more efficient use of root inoculation with SPB (and/or alternatively directly a product containing the siderophores) at the field scale, further investigations and knowledge, particularly at the mechanism scale, appear to be fundamental for the plant Fe nutrition context.

With respect to innovations through genetic modifications, there is no evidence in literature describing programs to improve the Fe-solubilizing traits of bacteria through biotechnological approaches.

## 4 Microbiome engineering-based approaches in biofertilizer development

Despite the well-documented beneficial effects of using biofertilizers based on single-strains or bacterial consortia, their adaptability to agricultural practices cannot be guaranteed, mainly because of their limited persistence in soil over time (Mitter et al., 2021). Nowadays, different microbiome engineering-based approaches involving the manipulation of microbial communities have emerged as a promising technology to promote synergistic interactions that can provide collective benefits to the host plant. These approaches include the creation of synthetic communities (*SynComs*) and the host-mediated microbiome engineering (HMME) (Yang et al., 2023). The key advantage of these approaches is not only the expected improvement of nutrient availability in the rhizosphere (with the consequent decrease in chemical fertilizer supplementation with the related environmental benefits), but also the positive effects of the rhizosphere microbiome to the plant health, amplifying the plant’s capability to cope with biotic and abiotic stresses.

### 4.1 The case study of biofertilizer based on synthetic communities

Synthetic microbial communities (*SynComs*) refer to artificial microbial consortia designed to recreate the core microbiome of a specific plant (Mitter et al., 2021). The synthetic consortium is normally engineered with three or more strains used to get a more realistic understanding of the interactions between microorganisms, plants, and the environment (Marín et al., 2021). According to their size, *SynComs* can be classified as a low- or high-complex consortia. Low-complex *SynComs* (<10 strains) are easier to design and can reduce costs and steps when scaling up bacterial growth in industrial processes. However, the small size of these consortia may not be taxonomically representative, thus neglecting important associations which are critical at the functional level. On the other hand, high-complex *SynComs* (>10 strains) can more efficiently mimic the autochthonous community in the rhizosphere with better chance of keeping associations intact, despite the limitations on their design (Mitter et al., 2021).

Regarding the use of *SynComs* as a potential strategy to develop ecology-based biofertilizers that enhance nutrient acquisition of plants, some studies have been carried out on this area. For instance, (Kaur et al., 2022) used a low-complex *SynCom* (4 strains) to investigate its impact on soil nutrient availability and growth of cotton plants. They found an increased nitrate availability in soils linked not only to the presence of the N-fixing *Brevibacterium* in the *SynCom*, but also to the enrichment of Cyanobacteria members induced by the consortium itself. Furthermore, after observing that *indica* rice recruited a higher proportion of bacteria related to N cycle as compared to *japonica* rice, Zhang et al. (2019) designed both a high-complex *SynCom* using16 *indica*-derived strains and a low-complex one using three *japonicum*-derived strains. The effects of both *SynComs* on the growth and N nutrition of IR24 rice plants were investigated and it was observed that the *indica*-derived high-complex *SynCom* was able to improve the transformation of organic N into NO_3_^−^ and NH_4_^+^ favoring, thus, in this crop higher values of N use efficiency (NUE). This result is perfectly coherent with historical observations of the higher NUE in *indica* varieties of rice than in the *japonica* ones (Hu et al., 2015).

Concerning the possible application of *SynComs* concept to plants growing in soils characterized by variable P availability, Finkel et al. (2019) designed a high-complex consortium encompassing 185 strains. Interestingly, in this experience a clear link between the presence of the genus *Burkholderia* and the efficient use of the P sources has been highlighted. In particular, plants colonized by a *SynCom* lacking genus *Burkholderia* displayed a higher P allocation at the shoot level than those inoculated with the complete consortium containing *Burkholderia* strains. These results highlight that the rhizosphere of plants can be also colonized by latent opportunistic microbial competitors, which, under a nutrient shortage, may be capable to further exacerbate the nutritional disorder of the host plant. Despite these observations, the *SynComs*, once inoculated in plants exposed to a nutrient shortage, have always proven themselves efficient in bringing benefits to the host plants. Similarly, (Harbort et al., 2020) developed a 115-strains *SynCom* to remediate Fe deficiency conditions in *Arabidopsis* plants. The research highlighted that the plant biosynthesis of coumarins and their exudation were required for *SynCom*-mediated rescue of plant development under Fe limitation. Interestingly, the same *SynCom* was not able to induce an over-accumulation of Fe, suggesting that the aiding activity of the consortium could be a plant-driven phenomenon, whose control was guaranteed by the root release of specific exudates. Overall, these pioneering studies, despite being at their beginning, clearly demonstrate the potential high functionality of the *SynComs* in enhancing the rhizosphere availability and the root acquisition of essential nutrients, yet overcoming some of the restraints of the single-strain biofertilizers currently available in the market.

### 4.2 The case study of host-mediated microbiome engineering

Plants generally influence the composition of the microbial community inhabiting their rhizosphere, where some microbial members are specifically recruited by the host, while other assemble opportunistically (Pantigoso et al., 2022). Hence, host-mediated microbiome engineering (HMME) is a biotechnological approach that uses the plant host to indirectly select specialized microbiome assembly colonizing the rhizosphere and, there, able to enhance plant growth, nutrient acquisition, and resilience to stresses (French et al., 2021). In some cases, such type of microbial selection has happened unintentionally after hundreds of years of domestication or during plant breeding programs, which led to the evolution of wild plant genotypes and, consequently, provoked an optimization in the rhizosphere microbiome of subsequent plants (Escudero-Martinez and Bulgarelli, 2023). For instance, corn is one of the most farmed and fertilized crops in the world, and its domestication has led to the recruitment in the rhizosphere of N-cycling functional groups as compared with ancestral corn genotypes (Favela et al., 2022). Moreover, it has been observed that, during nutrient starvation, corn (*Zea mays*) plants have evolved the ability to synthesize and release a variety of exudates into the rhizosphere. These organic compounds may have not only a role in influencing the biogeochemical cycles of nutrients in the rhizosphere, but also in promoting the recruitment of specific plant-beneficial taxa of microorganisms. In this context, it is interesting to mention the development of aerial roots secreting a huge amount of carbohydrate-rich mucilage observed in plants of a maize landrace grown under conditions of limited N availability (i.e., very limited or no fertilization) (Deynze et al., 2018). The authors described that the high levels of N fixation observed were supported by the abundant secretion of the mucilage, which favored the assembling of a complex N-fixing microbiome by roots. Similarly, under N deprivation, (Yu et al., 2021) observed that the flavonoids-releasing roots of maize plants specifically recruit in the rhizosphere bacteria of the taxon *Oxalobacteraceae*, that, in turn, facilitates N uptake through modulation of lateral root development. Based on these experiences, it appears evident that the reintroduction of alleles coding for beneficial compounds into crops by using HMME approaches could be a promising step toward the improvement of positive interactions between the plant rhizosphere and its microbiome.

Moreover, the increasing advances in plants-applied omics techniques has led to the identification of biosynthetic gene clusters (BGCs) responsible for the production of important secondary metabolites (e.g., terpenes, alkaloids, benzoxazinoids, cyanogenic glucosides, and polyketides). It is well known that these compounds are considerably involved in the assembly of rhizosphere microbiome (Polturak et al., 2022). Therefore, plant genetic transformations targeted to these BCGs clusters by using HMME based on genetic techniques, can represent a promising and futuristic step to reinforce the ability of plants to recruit specific beneficial microbiota. However, further research in this host-mediated microbiota selection by roots is still necessary. In particular, different aspects, like the choice of host trait, transferability of the recruited microbiome, and the environment component that can interfere with the selection of the most promising microbiome, might be considered and deeper addressed (French et al., 2021). Therefore, understanding the molecular and biochemical mechanisms governing the interactions between plant host and its associated microbiome, especially under nutritional disorders, would allow the design of crops with ability to produce/select “their own biofertilizers,” by reshaping specialized microbiomes that efficiently modulate (in their favor) the biogeochemical cycles of the nutrients in soil.

## 5 Current scenarios and future perspectives for PGPR-based biofertilizers

**Figure 2:**
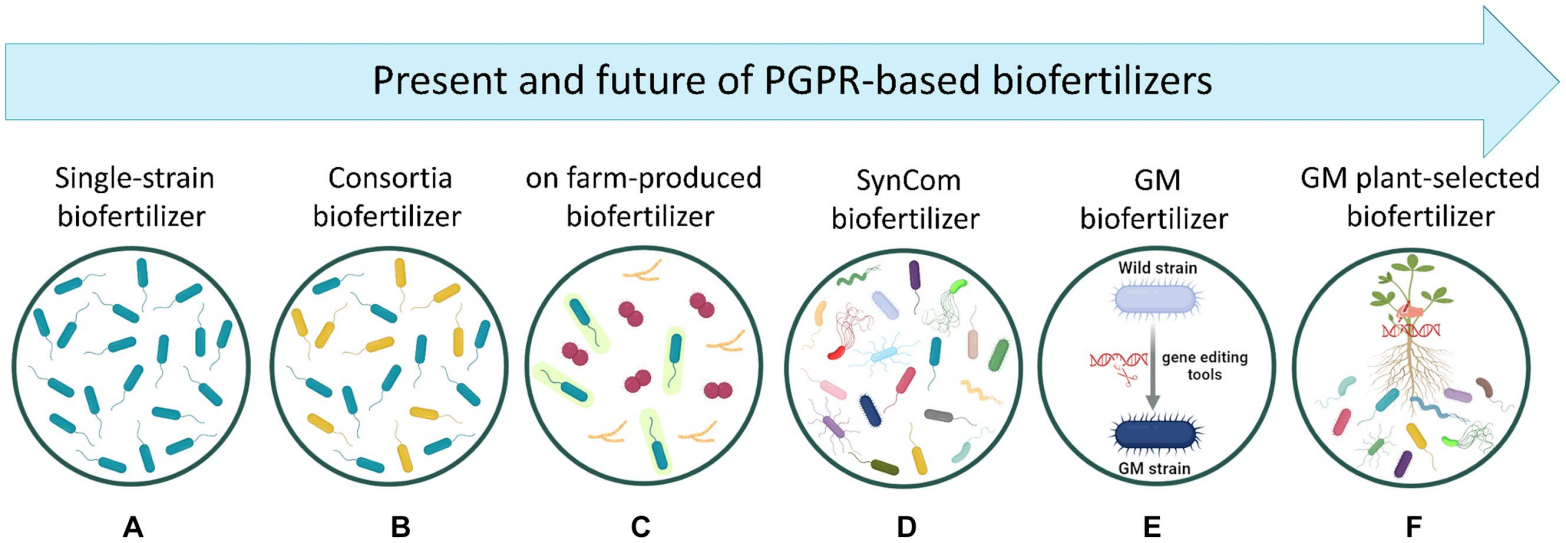
Representation of the present and future of PGPR-based biofertilizer development. **(A)** Biofertilizers based on a single bacterial strain. **(B)** Biofertilizers containing multiple strains (two or more) which are selected by considering their ability in enhancing plant uptake of soil nutrients. **(C)** Biofertilizers produced by farmers in their own farms by using rudimentary bio-factories without appropriate control of contaminations, which may result in highly contaminated, non-effective products. **(D)** Biofertilizers containing multiple strains which are selected after analyzing the diversity profile of plant microbiome. Thus, *SynComs* are designed to recreate the core microbiome containing key microbial taxa carrying essential functional genes for the host plant. **(E)** Genetically modified biofertilizers where bacterial genes are modified with gene editing tools (e.g., CRISPR/Cas9, RNAi) to improve plant growth and nutrition. However, the incorporation of these products into farm systems remains controversial since their efficacy, survivability, and environmental hazards are not well understood (Qiu et al., 2019). **(F)** Genetically modified plants driving the selection/recruitment of PGPR biofertilizers. Created with BioRender (https://biorender.com/).

The global biofertilizer market has to date an estimated value of USD 3.1 billion and it is projected to increase to USD 7.0 billion in 2030 (markets&markets). Currently, most of the biofertilizers available in the market are based on either a single PGPR strain with single or multiple PGP activities, or microbial consortia with multiple PGP activities (Figures 2A,B). Both kinds of products have shown success in promoting plant growth and improving soil physiochemical properties at a relatively low production cost. In this context it is interesting to note that *Bradyrhizobium*-based biofertilizers applied to soybean plants are probably the most successful example of these products across the world, fully supplying the crop’s demand of N in countries like Brazil (Santos et al., 2019). However, the efficacy of these biofertilizers often shows disparities due to (i) environmental variability, (ii) the intense competition with native microbial communities in the soil, and (iii) the application methods. Amazingly, due to the outstanding effects observed after the use of PGPR-based biofertilizers, some farmers have tried to produce their own biofertilizers using rudimentary bio-factories in a process called *on-farm* production (Figure 2C). This process consists of using makeshift equipment and infrastructure, including fermenters, open tanks, or even water tanks, without appropriate control of contaminations, which can lead to inconsistencies in production quality and efficiency (Bocatti et al., 2022). Therefore, beyond the admirable intention of these farmers, this *on-farm* practice represents a real threat for the entire agriculture in a general sense since plant, animal, and human pathogens have been predominantly found in such products, with non-effective results for plant growth and health (Bocatti et al., 2022). This aspect becomes even more relevant if the concept of One Health and its application in every context that concerns our planet, are considered.

Following the line of future possibilities in the development of high-performing biofertilizers, the design of *SynComs* represents certainly an innovative and promising technology (Figure 2D), as already detailed in the previous section. However, when producing biofertilizers based on these *SynComs*, some challenges shall be considered. One of them pertains to the ability of designing effective *SynComs* with a minimal number of bacteria. In this regard, it should be noted that for commercial uses, the industrial production of multi-strain biofertilizers is commonly performed by separately fermenting different batches and mixed them afterwards. Thus, it appears clear that, to scale up bacterial growth at industrial level, theoretically one fermenter per bacterial strain is required. However, if this approach were also applied to the production of high-complex *SynComs*, its economic and structural non-sustainability appears evident. In this scenario, the selection of bacterial strains displaying multifarious and synergistic traits is urgently needed to ensure a feasible and simplified way to produce *SynCom*-based biofertilizers.

In recent times, it became possible the use of genetic engineering approaches to optimize bacterial genes, thus providing the chance to set up eco-friendly biofertilizers improved in their capacity to ameliorate the edaphic conditions of the crops’ rhizosphere (Figure 2E). The genetic editing on targeted PGPR genes holds the potential of being fast and reasonably effective, due to the direct introduction of specific traits into well-characterized bacteria. Therefore, in the field of PGPR-based biofertilizers, genetic engineering stands as a promising strategy by which a wild-type PGPR strain can be optimized to overexpress the molecular machinery underlying N, P, K, S, Fe, and/or Zn acquisition for itself and the host plant. In fact, the manipulation of *nif*, *fix*, and *nod* genes in N-fixing bacteria have been shown to improve their symbiotic or associative performance in chickpea plants, leading to an increased N content of the crop tissues and yield (Silva et al., 2019). One other factor that could be exploited by using genetic manipulation is the insertion of genes related to PGP traits (one or more) into a bacterium that shows only one PGP mechanism. This approach may avoid the current need of mixing two bacterial strains when used in biofertilizer formulations. A hypothetical example of such types of transformation could be represented by a natural N-fixer that has been genetically strengthened with the ability to solubilize P and/or produce Fe-chelating compounds. Considering that for many years, engineering PGP traits has mostly focussed on N fixation and P solubilization, it is critical to enhance our knowledge about the molecular and functional mechanisms behind agronomically potential bacteria including KSB, SOB, and ZnSB to perform the most appropriated genome editing strategies toward more effective biofertilizers. Nevertheless, it cannot be ignored that the introduction into the environment of genetically manipulated bacterial strains might represent a potential risk of genetic pollution (i.e., horizontal genes transfer toward autochthonous microbiomes).

Besides the genetic modification of PGPR, another promising approach could be represented by engineering plants to either (i) increase their efficiency in nutrient acquisition and use, or (ii) recruit at the root level a more efficient microbial community. Considering the first point, and despite the genetic challenge, the development of plants able to directly fix atmospheric N without the help of bacterial partners would represent a feat without precedents, not only for alleviating adverse effects of synthetic N-fertilizers but also for the economic benefits derived from the higher harvest in system with low external inputs. In this regard it is interesting to note that current attempts are using the yeast *Saccharomyces cerevisiae* as model organism for initial testing of the functionality of eukaryotic nif proteins. In this attempt, chloroplasts and mitochondria are selected as candidate compartments for nitrogenase assembly and functioning (Burén and Rubio, 2018). With reference instead to the second point, plants could be also manipulated to select a specific rhizosphere microbiome capable of improving the nutrient acquisition process (Figure 2F). In this respect, it is well known that different plant species with contrasting physiologies and traits can shape different microbial community structures via root exudation. Therefore, by identifying exuded molecules related to the signaling and recruitment of specialized biofertilizing microbial communities, plant genes encoding the production of these exudates could be potentially manipulated. To achieve this objective, a deeper understanding of the mechanisms underlying root exudation patterns under different nutritional status and environmental conditions is needed. In this context, the integration of multiple omics approaches including metabolomics, ionomics, genomics, and transcriptomics could help identifying and deciphering the key components necessary for successful plant manipulation to efficiently “self-select” the best biofertilizing microbiome.

## 6 Common scepticisms about biofertilizers: from the lab to the field

Despite their potential, there have been ongoing discussions and doubts regarding the overall efficacy of PGPR-based biofertilizers in enhancing plant growth and increasing crop yields in real-world field conditions. This skepticism arises from the significant gap between the effects demonstrated by PGPR-based biofertilizers in laboratory or greenhouse experiments and the results found in field trials. While controlled experiments offer precision and reproducibility (Zuluaga et al., 2020; Scagliola et al., 2021), they often fail to capture the complex interactions between PGPR, plants, and soil microbiota present at field scale (Thilakarathna and Raizada, 2017; Meyer et al., 2019). Moreover, several reports suggest that the viability and proliferation of PGPR-based biofertilizers introduced into fields vary and are greatly influenced by environmental factors such as temperature, rainfall, and soil composition, as well as interactions with the host plant and indigenous microorganisms (Strigul and Kravchenko, 2006; Owen et al., 2015; Antoszewski et al., 2022). In addition, agronomical practices can significantly impact the composition of crops’ rhizosphere microbial community by modifying plant metabolism (Cesco et al., 2021). Similarly, these practices influence the epiphytic microbial community on fruits, which is affected by the plant’s nutritional state (Valentinuzzi et al., 2021). These findings underscore the complexity of the interactions among soil, microbes, and crops in the field, that may partly explain the varying levels of permanence and efficiency observed in the application of biostimulants.

In a meta-analysis conducted by Schütz et al. (2018), 171 peer-reviewed field studies were evaluated to assess the impact of biofertilizers on crop yield and nutrient use efficiency of N and P. The authors found that biofertilizers performed best in dry climates, showing a yield increase of 20.0%, compared to a 14.9% increase in tropical climates. Additionally, the yield response to biofertilizers was generally small at low soil P levels, but their efficacy improved with higher soil P levels, demonstrating the high variability of microbial biofertilizers under different field and environmental conditions. Similarly, a meta-analysis of 28 research papers examined the effectiveness of different rhizobial inoculants on soybean traits under field conditions (Thilakarathna and Raizada, 2017). It was shown that the effectiveness of the inoculants differed in terms of nodule count (−28 to +178 nodules) and grain-N yield (−6 to +176%) compared to uninoculated controls. This variability was attributed to various factors, including soybean genotype, interactions between soybean and rhizobia strains, inoculant formulation, concentration, and method of application. Adding to this complexity, research by Pandey et al. (1998) demonstrated that bacterial biofertilizers effective in subtropical climates struggled in temperate alpine regions due to their inability to establish and survive in lower temperatures. Furthermore, Sanz-sáez et al. (2015) found that inoculating soybean with *Bradyrhizobium japonicum* did not enhance photosynthesis, growth, or seed yield under ambient or elevated CO_2_ levels in the field, likely due to competition from native rhizobia.

**Figure 3:**
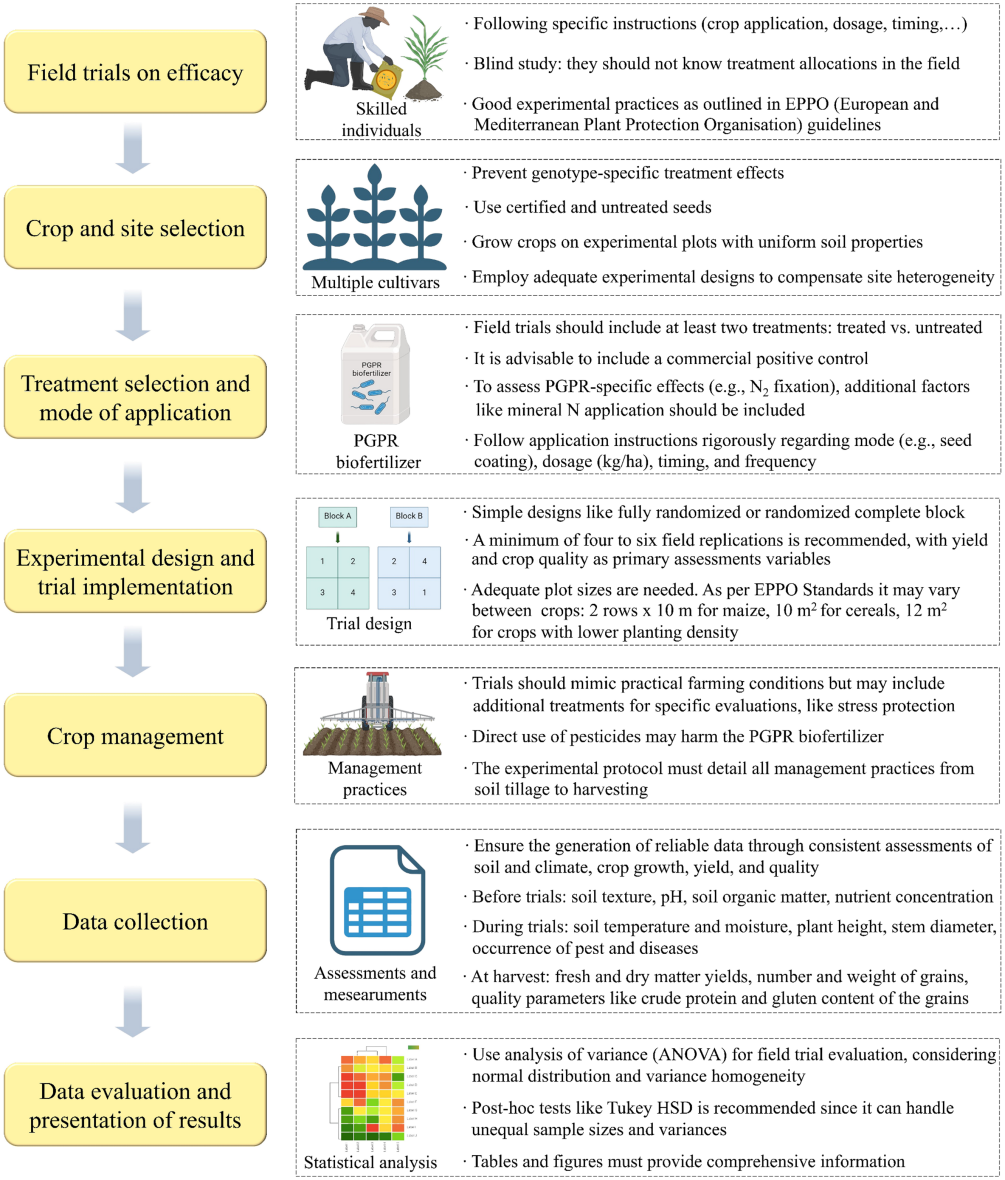
Guidelines for PGPR-based biofertilizers field trial design and implementation as proposed by Neuhoff et al. (2024).

In scientific literature, the prevalence of failed field experiments is likely considerable, yet such instances are seldom disclosed. This discrepancy primarily stems from the scientific community’s focus on innovation over the repetition and validation of previously published studies (Bashan et al., 2020; O’Callaghan et al., 2022). Moreover, the lack of standardized protocols for isolating, characterizing, and formulating PGPR strains exacerbates this issue, hindering the progression of biofertilizers from development to their ultimate field application. Standardized protocols would ensure consistency in application methods, such as the concentration of inoculants, timing, and techniques (e.g., seed coating versus soil drenching) allowing researchers and farming communities to draw more definitive conclusions about the efficacy of biofertilizers (Khan et al., 2023). Recognizing this need, Neuhoff et al. (2024) recently proposed a comprehensive set of standards for agronomic field trials, summarized in Figure 3, aimed at evaluating the efficacy of PGPR-based biofertilizers in arable crops within temperate climates. Thus, by adopting standardized protocols, the scientific community can address the gap in reliable field data, ultimately improving the progression of biofertilizers from experimental stages to widespread agricultural use. This approach will not only enhance the credibility of biofertilizer research but also support farmers in making informed decisions about their use, fostering broader acceptance and implementation.

## 7 Conclusion

It is evident that in the next future the current agriculture must face and meet important challenges whose success should consider the long-term environmental sustainability in a context of One Health paradigm and, properly of these last few years, the rapidly changing climate conditions. To achieve these objectives, all the tools and approaches that can contribute appear to be of crucial importance. The possibility of acting at a localized level at the rhizosphere (*precision farming*), allowing a more efficient exploitation of the endogenous nutrient resources already present in the soil as the PGPR-based biofertilizers seem to do, is certainly among the promising tools to contribute to the achievement of the general aim of a smarter and more sustainable agriculture. This review highlights how single strain and *SynComs* PGPR can impact soil fertility and nutrient availability for crop plants, how they influence the essential nutrient mobilization and uptake, and evaluates their effectiveness in controlled and field conditions. It also addresses skepticism about field results versus controlled experiments, advocating for standardized protocols and innovative approaches to improve biofertilizer performance. From this perspective, the potential of PGPR-based biofertilizers is evident, yet significant developments are needed regarding both the bacteria and the crops to optimize their use, especially in soils with altered fertility. Integrating advanced omics techniques, closely aligned with agricultural rhizosphere conditions, with cutting-edge bioengineering approaches could provide valuable insights. This integration is essential for advancing agriculture toward greater sustainability and resilience.

## Author contributions

MA: Conceptualization, Data curation, Investigation, Visualization, Writing – original draft, Writing – review & editing. RF: Conceptualization, Data curation, Investigation, Visualization, Writing – original draft, Writing – review & editing. SC: Conceptualization, Data curation, Funding acquisition, Visualization, Writing – original draft, Writing – review & editing. YP: Conceptualization, Data curation, Funding acquisition, Visualization, Writing – original draft, Writing – review & editing.

## Footnotes

^1^ https://sdgs.un.org/goals

^2^ http://www.marketsandmarkets.com
